# Stem Cell Transplantation for Multiple Sclerosis: A 2023 Review of Published Studies

**DOI:** 10.7759/cureus.47972

**Published:** 2023-10-30

**Authors:** Ali Msheik, Farah Assi, Faten Hamed, Ali Jibbawi, Anna-Marina Nakhl, Anthony Khoury, Rami Mohanna, Teddy Gerges, Rami Atat

**Affiliations:** 1 Neurological Surgery, Faculty of Medicine Lebanese University, Hadath, LBN; 2 Infectious Diseases, Faculty of Medicine Lebanese University, Beirut, LBN; 3 Pharmacology, Lebanese International University, Beirut, LBN; 4 Pediatric Medicine, Saint Georges Hospital, Beirut, LBN; 5 Medicine and Surgery, Faculty of Medicine Lebanese University, Beirut, LBN; 6 Medicine and Surgery, Faculty of Medicine Saint-Joseph University, Beirut, LBN; 7 Anesthesia, Winchester Anesthesia Associates, Boston, USA; 8 Neurology, Faculty of Medical Sciences Lebanese University, Beirut, LBN; 9 Neurology, Al Zahraa University Medical Center, Beirut, LBN

**Keywords:** secondary progressive multiple sclerosis (spms), primary progressive multiple sclerosis, relapsing-remitting, autologous hematopoietic stem cell transplantation, chemistry of multiple sclerosis

## Abstract

This comprehensive literature review underscores the potential of stem cell transplantation (SCT) as a therapeutic intervention for multiple sclerosis (MS). By amalgamating evidence from various sources, including randomized controlled trials (RCTs), observational, retrospective, and comparative studies, this review offers a holistic understanding of SCT's effectiveness, safety, and feasibility in diverse contexts of MS management.

SCT has shown promise in mitigating disease activity and progression, particularly in relapsing-remitting MS (RRMS). RCTs like the high dose immunoablation and autologous hematopoietic stem cell transplantation in MS (ASTIMS) versus mitoxantrone therapy in severe multiple sclerosis and multiple sclerosis international stem cell transplant (MIST) trials reveal SCT's capacity to reduce new lesion occurrences and inflammatory activity. However, variability exists in disability score improvements among these studies. Observational and retrospective investigations further affirm SCT's potential, highlighting decreased relapse rates, enhanced expanded disability status scale (EDSS) scores, and a noteworthy proportion of patients achieving no evidence of disease activity (NEDA).

The initial literature search using all of the search items produced a total of 3,636 articles. After title, abstract, and article type screening and article retrieving, 147 articles were assessed for eligibility using the inclusion criteria. At the end of the literature search, 37 articles met the eligibility criteria. They were included in our review according to preferred reporting items for systematic reviews and meta-analyses (PRISMA) guidelines.

Patients treated with hematopoietic stem cell transplantation (HSCT) present lower progression and relapse rates, suppression of inflammatory activity, and a greater reduction in T2 lesions on MRI than those treated with disease-modifying therapies (DMTs). In summary, while SCT presents promise as a therapeutic option for MS, its deployment should be tailored to individual patient characteristics, disease stages, and responses.

## Introduction and background

Overview of multiple sclerosis

Multiple sclerosis (MS) is a neurological chronic disorder that affects the central nervous system (CNS) by impairing communication between nerve cell fibers [1]. Auto-immune antibodies attack myelin in the CNS resulting in demyelination of neurons’ axons along with inflammation, gliosis, and neuronal loss. Currently, MS disorder prevalence is 2.8 million people worldwide predominantly in females with a ratio of 2-3:1 compared to males [2]. MS prevalence varies greatly between countries, from high levels in North America (especially Canada) and Europe (>100/100,000) to lower rates in Eastern Asia and sub-Saharan Africa (around 2/100,000) [3].

Inflammation, demyelination, reactive gliosis, and neuroaxonal damage are the key features of its pathology. Although the early course of the disease doesn’t cause much damage to axons and neurons, mainly damaging the myelin sheath, gradual neuroaxonal loss happens with ongoing disease, correlating with patient disability. Inflammatory lesions within the CNS are hallmarks of the disease and result in neuronal demyelination alongside axonal damage and multiple plaque formation in the brain and spinal cord [1-3]. These lesions are caused by infiltration of peripheral immune cells into the brain and spinal cord. Monocytes and macrophages, components of innate immunity, stimulate the migration of T-cells across the blood-brain barrier (BBB), resulting in its injury. An injured BBB permits the entry of systemic immune cells. Therefore, macrophages and CD8+ T-cells predominate the infiltrate at first, and lower numbers of CD4+ T-cells, B cells, and plasma cells may be present. With the development of the disease, B cells and plasma cells’ proportion increases while T-cell composition remains stable. The formation of plaques is mainly due to the chronic activation of microglia and macrophages and is involved in the loss of myelin sheaths and oligodendrocytes.

Clinically, MS symptoms majorly occur in young adults aged between 20 and 40 years old [1]. Neurological symptoms (vision problems, muscle weakness, bladder incontinence, and cognitive dysfunction) may vary leading to disability and further compromising patients’ everyday lives if no treatment is administered. Additionally, symptoms may vary depending on lesion location. Focused treatment must achieve improvement in key areas like relapse management, disease modification, and symptom management. There are four main types of MS, based on disease course: clinically isolated syndrome (CIS), relapsing-remitting MS (RRMS), primary progressive MS (PPMS), and secondary progressive MS (SPMS). Any part of the CNS may be involved resulting in a wide spectrum of symptoms ranging from cognition, motor, gait, sensory, and visual to bladder and bowel dysfunction [1].

Symptoms associated with the acute onset of partial transverse myelitis are one of the most common clinical presentations of MS. A typical involvement of the dorsolateral cord results in sensory symptoms. The extent of the lesion will determine whether symptoms are unilateral or bilateral. In PPMS, motor weakness, spasticity, and gait difficulty are usually dominant over sensory manifestations. Patients may also suffer from optic neuritis which typically presents with acute, unilateral, painful decrease in visual acuity. Additionally, eye movement is accompanied by mild to moderate pain. Diplopia is the most common brainstem manifestation due to internuclear ophthalmoplegia and may be bilateral. Facial weakness or loss of sensation may present alone or may accompany eye movement abnormalities. A lesion along the vestibular pathways results in vertigo. A cerebellar lesion may cause ataxia. Cognitive impairment is common in all MS phenotypes and begins early in the disease. Other unquantifiable symptoms that can have a major effect on patients’ quality of life include fatigue, depression, and sexual dysfunction [1].

Diagnostic criteria are based on clinical presentation accompanied by imaging that is consistent with MS. Cerebrospinal fluid (CSF) analysis is beneficial in cases where the diagnosis is not entirely clear [4].

Overview of stem cell transplantation (SCT)

SCT is a procedure in which a patient receives healthy stem cells to replace damaged stem cells [5]. Healthy stem cells can be taken from bone marrow, peripheral blood, or umbilical cord blood. This procedure produces a new immune system that appears tolerant and no longer attacks the central nervous system (CNS). It uses chemotherapy to remove the harmful immune cells and then rebuilds the immune system using new stem cells [5]. There are three types of SCT:

Autologous

An autologous SCT uses the patient's own stem cells. An advantage of an autologous SCT is that the patient is getting his own cells back. Consequently, there is no risk that the engrafted cells will be rejected by the patient’s body and there is no risk that the immune cells from the transplant will attack healthy cells in the patient’s body known as graft-versus-host disease (GVHD), which is a concern with allogeneic transplants. Also, the risk of infection is lower as there is no ongoing post-SCT immune suppression used to prevent GVHD. However, although extremely rare, a graft failure can happen in autologous SCT. Graft failure occurs when bone marrow function does not return. The graft may fail to grow in the patient leading to the absence of red blood cells, white blood cells, and platelet production resulting in infection, anemia, and bleeding [5].

Allogeneic

Allogeneic SCT involves the use of stem cells from a donor that can be related or not related to the patient. Finding a matching donor is key to a successful allogeneic SCT. Donor registries are used to find the appropriate match through tissue typing that uses proteins called human leukocyte antigens (HLA) found on white blood cells and tissues. An advantage is that the donor can often be asked to donate more stem cells or even white blood cells if needed. One complication of allogeneic transplantation is that the patient’s body may reject the donated stem cells before they are able to engraft in the bone marrow (graft failure). Allogeneic SCT is fraught with higher rates of morbidity and mortality, including GVHD - an event that can range from mild to life-threatening risks of opportunistic infections from immunosuppressants used to prevent GVHD [5].

Syngeneic

Syngeneic SCT is a special type of allogeneic transplant in which the donor is an identical twin (or triplet) to the recipient. GVHD will not be an issue because the immune system recognizes the identical sibling’s stem cells as the patient’s own as a result of 100% HLA matching between the donor and the recipient. As with any type of transplant, there is a risk that the transplanted donor stem cells could die or be destroyed by the patient’s body before settling in the bone marrow [5].

Methods

Research Resources and Timeframe

**Figure 1:**
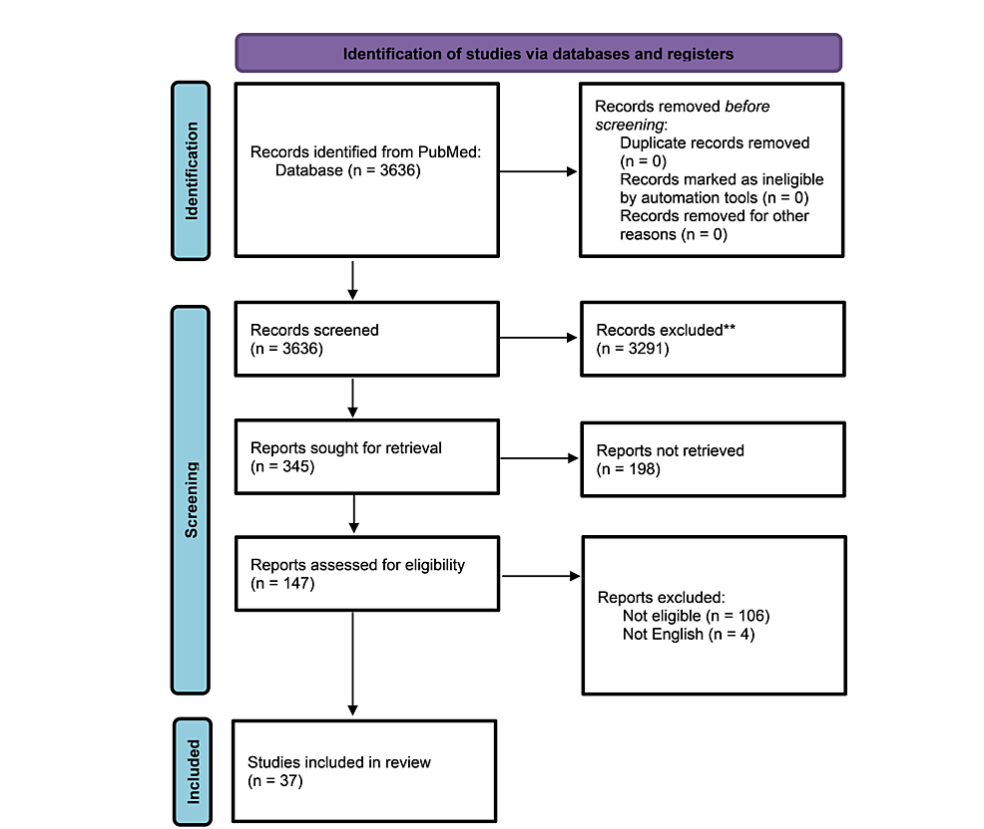
A preferred reporting items for systematic reviews and meta-analyses (PRISMA) flow diagram visually summarizing the selection process of the studies **exclusion criteria: articles that are literature reviews and irrelevant context.

A comprehensive literature review on the role of stem cell transplantation in MS treatment is essential due to the growing significance of this therapeutic approach in managing the disease. By analyzing existing literature, researchers and healthcare professionals can gain insights into the safety, efficacy, and long-term benefits of stem cell transplantation in alleviating MS symptoms and halting disease progression. By the application of the preferred reporting items for systematic reviews and meta-analyses (PRISMA) guidelines, a thorough review of published studies will not only provide a deeper understanding of the current state of stem cell therapies but also shed light on potential areas of improvement, challenges, and future directions for optimizing this innovative approach to MS treatment. The PubMed database was extensively searched in July 2023. The 27 included observational and retrospective studies assessing the efficacy of hematopoietic stem cell transplantation (HSCT) for the treatment of MS were published from 2002 until 2023, enrolled patients starting from 1991 until 2021, and involved a total of 2,002 subjects (Figure 1). The following terms were used in the search strategy: multiple sclerosis, MS, stem cell transplant, and HSCT. 

Eligibility criteria

Original articles, of type randomized controlled trials, observational, retrospective, and comparative, which evaluate and report the efficacy of hematopoietic stem cell transplant in the treatment of MS were included in this review. The eligible studies must be of the above-mentioned article types and must provide a measurement of the efficacy through any of MS clinical prognostic factors [enhanced expanded disability status scale (EDSS) change, progression-free survival (PFS), annualized relapse rate (ARR) or number of relapses, relapse-free survival (RFS), no evidence of disease activity (NEDA), confirmed disability progression or improvement, or MRI event-free survival (MFS)]. Nonrandomized and uncontrolled studies, case reports, reviews, articles that included populations other than MS, articles that included pediatric population, and articles not written in English were excluded from this review. We defined PICO as follows: Problem or study population (P): patients with MS; Intervention (I): HSCT; Comparison (C): Standard MS disease-modifying therapy (DMT) or immunological treatments when available; Outcome (O): Efficacy.

Out of 3636 articles identified from PubMed and after the screening, only 147 articles were assessed for eligibility, of which 106 were not eligible and six were non-English articles. Hence, only 37 articles were included in the review [5-39].

## Review

The initial literature search using all of the search items produced a total of 3,636 articles. After title, abstract, and article type screening and article retrieving, 147 articles were assessed for eligibility using the inclusion criteria. At the end of the literature search, 37 articles met the eligibility criteria and were included in our review according to PRISMA guidelines. Details about the effectiveness of SCT per type of study and type of MS, the safety and feasibility of SCT, and the future considerations are depicted in the subsequent sections. A summary of the articles is revealed in Table 1.

**Table 1:** Key points and outcomes of reviewed randomized controlled trials (RCTs), observational, and retrospective studies which aimed to evaluate the efficacy of hematopoietic stem cell transplantation (HSCT) in treating various stages of multiple sclerosis (MS). MS: multiple sclerosis; EDSS: expanded disability status scale; iEDSS: initial EDSS; fEDSS: final EDSS; MRI: magnetic resonance imaging; fMRI: final MRI; RRMS: relapsing-remitting MS; SPMS: secondary progressive MS; PPMS: primary progressive MS; PFS: progression-free survival; ARR: annualized relapse rate; Gd+: gadolinium-enhancing; PRMS: progressive-relapsing MS; RFS: relapse-free survival; MFS: MRI event-free survival; NEDA-3: no evidence of disease activity with no new MRI activity, no new clinical relapses, and no confirmed worsening of EDSS; T2W: T2-weighted; NRS: neurological rating score; PASAT: paced auditory serial addition test; SF-36: short form-36 quality-of-life score; RCT: randomized controlled trial; HSCT: hematopoietic stem cell transplant; NEDA-4: NEDA-3 with no brain volume loss 0.4% or more per year; CDP: confirmed disability progression; SDI: sustained disability improvement; CDA: continuous disability accrual; NEDA-2: absence of relapses and absence of EDSS worsening; OW: overlap weighting; PS: propensity score; CDP: confirmed disability progression; Gp: group; WFS: worsening-free survival.

Author, year	Type	Sample size	Age (y)	MS duration (y)	MS Stage	Follow-up duration (y)	iEDSS	Regimen	fEDSS	fMRI	Prognostic factor reported	Deaths	Notes
Mancardi et al., [6]	RCT Monocentric	9	36	10.5	RRMS (33%) SPMS (29%) SPMS with relapses (33%) RPMS (5%)	4	6.5	Intermediate intensity myeloablative		- T2W lesions: 79% reduction - 100% Gd+ lesions-free	- ARR: 0.19 - progression in 57%	0	
Burt et al., [7]	RCT Multicentric	55	35.6	5.25	RRMS (100%)	2.8	3.4	Intermediate intensity nonmyeloablative	At 1y: 2.36	At 1y: 68% decrease in volume of T2W lesions	- relapse at 2 & 4y: 7.69% & 15.4% rsp	0	NRS score decreased (improved) total short form 36-qality of life scores improved
Boffa et al., [8]	Observational Multicentric	- OW matched: 79 - PS matched: 69	37.5 (OW) 37.1 (PS)	12.1 (OW & PS)	SPMS (100%)	5.6 (OW) 6.8 (PS)	6 (OW) 6.5 (PS)	Heterogeneous	- EDSS (PS) improvement and maintained stabilization at 3y in 34.7% (At 5y: 18.7%)		OW: -ARR (2y): 0.025 - ARR (overall): 0.02 PS: -CDP-free (3y): 71.9% - CDP-free (5y): 61.7% - ARR (2y): 0.024 - ARR (overall): 0.02	1	EDSS (OW) change: (-0.017)/year maintained over 10 y EDSS (PS) change: (-0.013)/year maintained over 10 y longer time to CDP in (PS) HSCT arm
Mariottini et al., [9]	Retrospective Monocentric	31	39.3	13.7	SPMS (100%)	8.3	5.6	Intermediate intensity myeloablative	EDSS worsening-free survival: 45%		- RFS 100% at all time points - ARR (2y): 0.00 - PFS (3&5): 84% & 70% - NEDA-2 y5: 45% (y2 55%)	0	
Mariottini et al., [10]	Retrospective Monocentric	26	37	9	SPMS (100%)	8.25	6	Intermediate Intensity myeloablative	61.5% worsened at final follow-up	Activity completely suppressed (0%)	- PFS (at 3,5, & 10y): 48%, 42%, & 30% rsp - CDA free-survival: 74% at 5y - NEDA-3: 42% at y5 & 30% at y10 - ARR: 0	0	
Jespersen et al., [11]	Retrospective observational Monocentric	32 Gp A: 7 Gp B: 25	A: 46 B: 36	A: 12 B: 6	RRMS (100%)	A: 4 B: 3.3	A: 5 B: 3.5	Heterogeneous intermediate-intensity (Gp A: Myeloablative Gp B: non-Myeloablative)	At 2 years: A: EDSS improved in <14% B: EDSS improved in 24%		At 2 years: - RFS 77% (A 57%, B 83%) - WFS 79% (A 57%, B 85%) - MFS 93% (A 86%, B 96%) - NEDA-3 67% (A 43%, B 77%)	0	- better survival curves for B than A
Kalincik et al., [12]	Observational comparative Multicentric	- n= 167 Matched: Arm 1: 144 Arm 2: 146 Arm 3: 110	35.3 to 37.1	7.92 to 8.68	RRMS (100%)	3.78 to 4.08	3.5 to 3.86	Heterogeneous			ARR: - Arm 1: 0.09 - Arm 2: 0.10 - Arm 3: 0.09 30% reduction of disability progression in HSCT arms 1&2	1	HSCT-ARR significantly decreased from pre-treatment in all three groups Arm 1: final ARR superior to comparator Arm 2: final ARR marginally better than comparator Arm 3: final ARR did not show superiority
Zhukovsky et al., [13]	Observational Multicentric	69	30	6.4	RRMS (100%)	2.8	3	Intermediate intensity nonmyeloablative	EDSS improved by 1 point	MFS at 3y: 93%	At 3y: - NEDA-3: 88% - ARR: 0.04	0	
Boffa et al., [14]	Retrospective Monocentric	HSCT= 25	32.1	9.5	RRMS (100%) (Aggressive)	4.3	6	Intermediate Intensity myeloablative	EDSS improved	- MFS: 85%	- NEDA-3: 75% - RFS: 84% - CDP: 88% - ARR 0.05 at 3 y		MRI activity significantly less post-HSCT Time to retreatment in HSCT arm significantly longer
Kvistad et al., [15]	Observational Monocentric	30	29.5	4	RRMS (100%)	2.2	3	Intermediate Intensity nonmyeloablative	- Improved: 43% - Stabilized: 50% - Progression: 7%	- new T2W lesions in 10%	- NEDA-3: 83% at 2.2 y	0	Clinical relapses post-HSCT: 10%
Giedraitiene et al., [16]	Observational Monocentric	Efficacy = 13 Total= 24	37.8	8.6	RRMS (100%)	1.9	5.9	Intermediate intensity nonmyeloablative	At 1 y: - Improved: 23.1% - Stable: 76.9%	No new MRI activity	At 1 and 2 y: - ARR 0.2 and 0.3 (89% reduction) - SDI at 1 y in 76.9%	0	
Mariottini et al., [17]	Retrospective Monocentric	HSCT= 11	35	13	RRMS (100%)	3	3.25	Intermediate intensity myeloablative	3.75 EDSS improvement in 44% at 3 years		3 years - NEDA-3: 54.5% - ARR 0.00	0	none in HSCT progressed to progressive MS long term disability improvement significantly higher 7 patients crossed-over to receive HSCT
Tolf et al., [18]	Observational Monocentric	10	27	2.33	RRMS (100%)	10	6.5	heterogenous (intermediate and low intensity)	1.75		- NEDA-4: 50% - NEDA-3 (5y): 70% - resolved MS: 30%	0	
Kvistad et al., [19]	Retrospective Observational Multicentric	104	30	5.8	RRMS (100%)	3.3	3	Intermediate intensity nonmyeloablative	_	- New MRI activity in 7.7%	- NEDA-3: 81%	0	
Comini-Frota et al., [20]	Observational Monocentric	5	33	11	RRMS (100%)	9	6	Low Intensity	At 5y: 4.5			0	
Burt et al., [21]	Observational Monocentric	507	37	6	RRMS (81.6%) SPMS (18.3%)	3	4	Heterogeneous	- 2.5 at 2y - at 5y - EDSS newly-diagnosed SPMS improved for 1y after HSCT then stabilized		- PFS (2,4 y): 93.13% & 89.6% - RFS (2;4 y): 95.9% & 91.3%	1	RRMS: PFS 4y 95% SPMS PFS 4y: 66% RRMS: RFS 80.1% at 5 SPMS RFS at 5y: 98.11% No difference in EDSS between different conditioning regimens
Burt et al., [22]	Observational Monocentric	145	37	5	RRMS (81.4%) SPMS (18.6%)	2	4	Intermediate Intensity nonmyeloablative	at 5y: 2.5	at 5y: - Gd+ lesions nb: 0.08 - T2W lesions volume: 5.74 cm³ (decrease by 33%)	at 4y: - PFS 87% - RFS 80% - NEDA-3 68%	0	NRS: from 74 to 85 at 5y 25-foot walk: from 6.22 sec to 4.35 at 5y 9-hole peg test: from 24.68 sec to 23.49 at 5y PASAT-3 score: from 71.69% to 90% at 5y SF-36 2y: from 45 to 64
Nicholas et al., [23]	Retrospective Multicentric	120	42.3	8.9	RRMS (48%) SPMS (33%) PPMS (18%)	1.75	6	heterogeneous	EDSS stable at 4y in 65%	MFS: 85% at 4y	At 4y: - PFS: 65% - ARR: 0.08 - NEDA-3: 53% (65% at 2y) - RFS: 87%	3	RFS-4y (RR): 77% high (≥5 g/L) paraproteinemia: associated with EDSS progression over 4 years
Muraro et al., [24]	Retrospective Multicentric	281	37	6.8	RRMS (16%) PRMS (6%) SPMS (66%) PPMS (11%)	6.6	6.5	Heterogeneous			at 5y: - PFS 46% (RMS 73%) (PMS 33%)	8	PFS: better in younger age, progressive form, and receipt of < 3 prior treatments
Fassas et al., [25]	Retrospective Observational Multicentric	85	39	7	RRMS (4%) SPMS (70%) PPMS (26%)	1.3	6.5	Heterogeneous	14% showed improvement of EDSS	Active lesions 8%	PFS at 3y: 74% total PFS (PPMS): 66% PFS (RRMS & SPMS): 78%	5	Higher PFS in age< 40 (89% vs. 58%), and stage not PPMS
Mancardi et al., [26]	Retrospective Multicentric	74	35.7	11.2	RRMS (45%) SPMS (55%)	4	6.5	Intermediate Intensity Myeloablative	> 7y: Improvement: 27% Stable: 17% 56% progressed	Active lesions at 2y (Gd+): 8.3%	- PFS (5y): 66% RR PFS: 71% SP PFS: 62% - Relapse-free at 5y: 85%	2	5y-PFS higher in: age <40y (69%) vs. > 40y (52%), in RR stage (71%) vs. SP (62%), & MRI with Gd+ lesions at baseline (87%) vs. no Gd+ lesions (46%)
Burman et al., [27]	Observational Multicentric	48	31	6.3	RRMS (83.3%) SPMS (10.4%) PPMS (4.2%) PRMS (2%)	4	6	Heterogeneous (intermediate-intensity myeloablative & nonmyeloablative)	at 2y: RRMS: 3 PRMS: 6.5	5 patients had new active MRI lesions	at 5y: - PFS: 77% - RFS: 87% - MFS: 85% - NEDA-3: 68%	0	No prognostic difference between treatment with myeloablative vs. nonmyeloablative regimen
Chen et al., [28]	Retrospective Monocentric	25	37.3	4	RRMS (12%) SPMS (76%) PPMS (4%) PRMS (8%)	5	8	- Intermediate intensity myeloablative (n=24) - High intensity regimen in one case	- At 1^st^ year: 5.5 - At 6-7-8 years: 7 - EDSS score: 40% improved 28% stable 32% worsened	Active lesions in 17% (2/12)	- PFS (3, 6, & 9 y) follow-up: 74%, 65%, & 48%	2	
Casanova et al., [29]	Observational Multicentric	31	35.7	8.4	RRMS (71%) SPMS (29%)	8.	5.3	Intermediate intensity myeloablative	4.5	at 5y: - increase in nb of T2W lesions in 2 patients	- ARR: 0.05 (6-7 yr) - NEDA: 54.8%	0	92% reduction in ARR at 2y post-HSCT at 7y: 60% showed disability improvement, 40% stabilized
Krasulova et al., [30]	Observational Monocentric	26	33	7	RRMS (42%) SPMS (58%)	5.5	6	Intermediate intensity myeloablative			- PFS: 70.8% at 3y; 29.2% at 6y PFS (RRMS): 84.4% PFS (SPMS): 60% - ARR: 0 at 2y	0	- PFS: No significant difference between EDSS <6 and those with EDSS > 6 - Better PFS in disease duration < 5y, and in patients <35 years old
Häußler et al., [31]	Observational comparative Monocentric	19	35.1	5.4	RRMS (63.1%) SPMS (21.1%) PPMS (15.8%)	4.9	4.5	Intermediate intensity myeloablative	EDSS 1y-improvement: 35.7%	_	- NEDA-3: 62% (vs. 40.2%)	0	long term cognitive performance significantly improved in HSCT group
Das et al., [32]	Retrospective Multicentric	20	28	0.42	“Aggressive MS”	2.5	5	Heterogeneous	2	MRI activity: 15%	At 2.5y - NEDA-3: 85%	0	
Dayama et al., [33]	Observational Monocentric	20	31.5		RRMS (45%) SPMS (55%)	0.7	5.5	Intermediate Intensity nonmyeloablative	5.5		- PFS 1y: 100%	0	EDSS improvement in 36% (66.6% RRMS and 10% SPMS)
Frau et al., [34]	Retrospective Monocentric	9	38	10	RRMS (55.5%) SPMS (22.2%) PPMS (11.1%) PRMS (11.1%)	11 md	5.3	Heterogeneous	5.7	- new lesions in 66.7% - Gd+ lesions in 77.8%		0	Significant reduction in nb of relapses 2 y after HSCT (1.4) EDSS improved in 22.22%, stabilized in 22.22%, and worsened in 55.6%
Dahbour S et al., [36]	Open‐label prospective phase I/IIa	25 patients assessed for eligibility, 15 patients treated, 10 patients analyzed	Mean (±SD); 34.9 (±9.54) Range (A) Age (Y)(18; 54)	Mean (±SD); 9.6 (±2.91) Range (A)(4; 15)		1 year	5.1 (±1.73)	the highest reported dose of autologous MSCs into MS patients (93‐168*106) via the intrathecal route. It was followed a month later with the MS‐CM (15‐20 mL)	5 (±1.86)				
Violaine K. Harris et al., [37]	phase I, open-label single-arm study	20	49 (27–65)	19 (10–32)	SPMS 16 (80) PPMS 4 (20)	2 years	6.8 (3.5–8.5)	3 IT injections of MSC-NPs spaced 3 months apart	8 of 20 subjects had demonstrated at least a 0.5 point improvement in EDSS, with 4 of the 8 subjects showing an improvement of 2.0 or greater positive change compared with baseline.1 At the 2-year follow-up assessment, 7 of the 8 subjects showed continued improvement. Two subjects demonstrated sustained improvement of 2.0 or greater and 5 subjects demonstrated sustained EDSS improvement of 0.5 points One subject (12) who had previously demonstrated 1.0-point improvement between baseline and 6 months demonstrated a 0.5-point worsening from baseline at 2 years. In the remaining subjects, 6 showed continued stable EDSS throughout the course of the study, 2 subjects (10 and 13) continued to show disease worsening at all follow-up visits from baseline and an additional 2 study subjects (14 and 15) showed disease worsening at 2 years compared withthe 6-month visit		the timed 25- foot walk (T25FW)		This study provides Class IV evidence that for patients with progressive MS, IT administration of MSC-NPs is safe and effective. The study is rated Class IV because of the absence of a non–IT-MSC-NP-treated control group
Panayiota Petrou et al., [38]	double-blind RCT	48	47.5 ± 12.3	Disease onset (y) 12.70 ± 7.51	20 activea : 18 SPMS, 2 PPMS 28 non-active: 23 SPMS, 5 PPMS	1 year	3.0–6.5, mean : 5.6 ± 0.8	The study included two phases (cycles of treatment): in the first phase, three groups of patients were formulated after randomization: one group (group 1) was assigned to receive an intrathecal injection of 1 106 MSCs (MSC-IT)/kg of body weight, and an intravenous sham injection of normal saline. The second group (group 2) was treated with an intrathecal sham injection (normal saline) and an intravenous injection of 1 106 MSCs (MSC-IV)/ kg of body weight, and the third group (group 3: sham treatment) was treated only with normal saline (intravenously and intrathecally). In the second phase after 6 months, the treatment groups were crossed over and each one subdivided into two subgroups of eight patient	6.0 (5.87–6.5)	: 0.55 ± 1.03the sham-treated group, 0.17 ± 0.47 in the MSC-IT group, and 0.97 ± 1.93 in the MSC-IV group			
Boffa, James et al., [39]	observational, retrospective, multicenter cohort study	210 pateints	6.2(±5.0) years	11.0 (6.7)	(58%) had a relapsing-remitting (RR) phenotype of MS (RRMS), 86 patients (41%) had 133 secondary progressive (SP) MS and 2 patients (1%) had primary-progressive MS	Patients from 20 Italian MS centers who underwent transplant from 1997 to 2019 were identif	6(1-9),	BEAM+ATG regimen (74.8%), which includes BCNU (carmustine, 300 mg/m2 at day -6), cytosine-arabinoside (200 mg/m2) and etoposide (200 mg/m2) from day -5 to day -2 and melphalan (140 mg/m2) at day -1, followed by rabbit anti-thymocyte globulin (ATG) (3.75-5 80 mg/kg/day) at days +1 and +2; BEAM regimen as above described without rabbit ATG (4.8%); FEAM regimen (1.9%), substituting fotemustine (150 mg/m2 on days -7, -6) instead of BCNU in the BEAM regimen; CY+ATG regimen I (8.1%), containing CY (60mg/kg at day –3 and –2) followed by rabbit ATG (3.75 mg/kg/d at day +1 and +2); CY+ATG regimen II (4.8%), containing CY (50 mg/Kg/d at days -5 to day -2) and rabbit ATG (2.5 mg/Kg/d at day -4 and -2); Thiothepa+CY regimen (4.8%), consisting of thiothepa 10 mg/kg for 5 days and CY 50 mg/kg at day -3 and -One patient was transplanted with a conditioning regimen made of BCNU and 87 melphalan (0.5%) and one patient was transplanted with a conditioning regimen made of bortezomib, cyclophosphamide, dexamethasone and melphalan (0.5%). Anti-herpetic and anti pneumocistis jirovecii prophylaxes were performed with Acyclovir and Sulphamethoxazol Trimetoprim, respectively, according to centers protocols. After aHSCT, patients did not receive immune-based therapies unless they experienced clinical relapse, new lesions on MRI, or EDSS progression, based on decision by the treating neurologist	EDSS significantly reduced after aHSCT [p=0.001; mean EDSS change per year -0.09 (95%CI=-0.15 to -0.04%)].	the percentages of patients free of MRI inflammatory activity were 78.7% (71.1%-86.3%) at 5 years and 64.3% (52.7%-75.9%) at 10 years	disability worsening-free survival (95%CI) was 85.5%(76.9-94.1%) at 5 years and 71.3%(57.8-84.8%) at 10 years. In patients with progressive MS, disability worsening-free survival was 71.0%(59.4-82.6%) and 57.2%(41.8-72.7%) at 5 and 10 years, respectively	3	aHSCT prevents disability worsening in the majority of patients and induces durable improvement in disability in patients with RRMS. The BEAM+ATG conditioning protocol is associated with a more pronounced suppression of clinical relapses and MRI inflammatory activity

Evidence for the effectiveness of SCT in MS

Randomized Trials

Only two randomized controlled trials have been conducted that evaluate the efficacy and safety of HSCT in MS patients, both with methodological limitations. The autologous hematopoietic stem cell transplantation trial in MS (ASTIMS) is a phase II multicenter study conducted by Mancardi et al. who enrolled patients from 2009 to 2015 [6]. The ASTIMS study compared the efficacy of intermediate intensity myeloablative autologous hematopoietic stem cell transplantation (aHSCT), using the bis-chloroethylnitrosourea, carmustine (BCNU), etoposide, ara-C, and melphalan (BEAM) + anti-thymocyte globulin (ATG) regimen, vs. mitoxantrone (MTX) as the standard of therapy in the control group. The study has demonstrated the superiority of HSCT in reducing the appearance of new T2 lesions on MRI (79%). These effects were not only evident in the first year after therapy, but also remained upheld throughout the entire four-year follow-up. The efficacy of HSCT was also demonstrated by the suppression of inflammatory activity and relapse rate in this arm. However, an improvement in the EDSS score was not demonstrated in the HSCT arm, and progression was seen in 57% of the subjects.

The second study is a phase III multicenter randomized controlled trial (MIST) conducted by Burt et al. and enrolled a total of 110 patients from 2005 to 2016 [7]. The study employed intermediate-intensity non-myeloablative chemotherapy using cyclophosphamide + ATG regimen in the HSCT arm vs. DMT in the control group [7]. At five years follow-up, 9.71% of the patients in the HSCT arm experienced disease progression vs. 75.3% of the patients in the DMT group. Similarly, the relapse rates for the HSCT group were significantly lower than that in the DMT group at five-year follow-up.

Both RCTs have had certain limitations to consider. The ASTIMS study had a small sample size. As a consequence, it was converted into a phase II study rather than a phase III study as originally intended. Furthermore, the number of included patients with progressive forms of MS, which generally are less responsive, was relatively high. This might explain the absence of improvement or even stabilization in the disability score results in the HSCT arm. The sample size of the MIST study was relatively small compared to sample sizes in pharmaceutical-sponsored trials that established the efficacy of DMT. In addition, a certain number of patients have crossed over from the DMT arm to the HSCT arm due to their worsening EDSS score, which resulted in incomplete follow-up data for patients receiving DMT. This, however, did not affect the endpoints of time-to-progression, and time-to-relapse, nor NEDA. In regards to the DMT regimens, certain treatments (alemtuzumab, ocrelizumab, ofatumumab, cladribine) were not included in this study for reasons related to FDA approvals at the time of enrollment and concerning side effects.

Observational, Retrospective, and Comparative Studies

The sample sizes ranged from a minimum of five participants to a maximum of 507. Minimal median follow-up duration was 0.7 years and the maximal was 11. The conditioning regimens for the HSCT procedure differed across the studies. Nineteen studies used intermediate-intensity conditioning regimens, eight of which were myeloablative, six of which were nonmyeloablative (lymphoablative), and five of which used a combination of both. Only one study used a low-intensity regimen, and the seven remaining studies used heterogeneous conditioning regimens which included high, intermediate, and low intensity, with or without biological agents in some articles. The efficacy was evaluated using various prognostic tools over a period of time: EDSS change before and after the procedure, PFS, NEDA, RFS, ARR, and others.

SPMS

The recent observational study by Boffa et al. aimed to evaluate the efficacy of HSCT in halting the progressive phase and the time to progression in SPMS [8]. The study has clearly demonstrated better disability-free outcomes in SPMS patients treated by HSCT. In 2022, Mariottini et al. showed an EDSS worsening-free survival of 45%, alongside a significant reduction in the ARR to 0.00 post HSCT corresponding to a NEDA-2 of 45% at year five of follow-up, in the retrospective study that included a total of 31 SPMS patients treated with HSCT [9]. Another article published in 2021 by Mariottini et al. retrospectively including only SPMS patients (n=26) who were treated with HSCT has shown similar outcomes [10].

RRMS

Jespersen et al. evaluated the efficacy of HSCT in a cohort of 32 relapsing-remitting MS patients and compared the effectiveness of myeloablative and non-myeloablative intermediate-intensity conditioning regimens [11]. Comparing the nonmyeloablative regimen with the myeloablative, the cyclophosphamide (Cy) group reported better outcomes and significantly better survival curves than the BEAM group at the two-year follow-up.

Kalincik et al. recently conducted a multicenter observational comparative study evaluating the efficacy of HSCT in comparison to other DMTs [12]. The study has demonstrated the superiority of HSCT over fingolimod in blunting relapse rates. These results in comparison with natalizumab were only marginal. Treatment with HSCT demonstrated an early higher rate of recovery from disability compared to the DMTs in the first and second arms.

A similar degree of relapse suppression was observed in the multicentric observational study by Zhukovsky et al.. The study involved 69 RRMS patients, with a 6.4-year median duration of disease, a median baseline EDSS of 3, and a baseline ARR of 1.4, who were followed-up for a median duration of 2.8 years after HSCT [13]. The outcomes were compared to a sample of patients treated with alemtuzumab. At three years of follow-up, the median EDSS score decreased, and the ARR was significantly suppressed to 0.04 with a 93% MRI event-free activity, thus corresponding to an NEDA-3 of 88% at follow-up evaluation.

Likewise, Boffa et al. selected a population of “aggressive” MS patients and compared the efficacy of HSCT (n=25) with a cohort of patients treated with alemtuzumab (n=32) [14]. Similar to previous studies, the study demonstrated significantly better outcomes in the transplant arm at the end of the follow-up period [NEDA-3 75%, RFS 84%, confirmed disability worsening (CDW) 88%, MFS 85% ARR 0.05], as well as a longer time for retreatment, as compared to alemtuzumab arm.

Comparable results were reported by Kvistad et al. where a NEDA-3 of 83% after a median follow-up of 2.2 years was reported [15]. An EDSS score improvement was observed in 43%, and stabilization in 50%. MRI activity was absent in 90%.

An impressive 89% reduction in the ARR two years following transplant (from 4.6 to 0.5) was demonstrated by Giedraitiene et al. with 76.9% showing sustained disability improvement at one-year follow-up [16]. The EDSS score improved by 23.1% and stabilized by 76.9%, with complete blunting of the MRI activity.

Mariottini et al. designed a study to evaluate the efficacy of HSCT vs. a cohort of DMT following natalizumab cessation [17]. The resultant ARR after three years of follow-up was completely suppressed (0.00) in the HSCT and NEDA-3 was 54.5% vs. 0.67 and 11.5% in the DMT group. 44% showed improvement in the EDSS score in the HSCT and none progressed to progressive MS.

In the article by Tolf et al., albeit having a small sample size (n=10), 70% of the subjects reached NEDA-3 at year five of follow-up, and the remaining 30% achieved disease resolution [18]. The final EDSS score decreased from a median of 6.5 to a median of 1.75. NEDA-4 was 50%. Kvistad et al. evaluated the safety and efficacy of HSCT in a cohort of 104 RRMS patients [14]. The resultant NEDA-3 at a median follow-up period of 3.3 years was 81% [19]. Comini-Frota et al. followed five patients with RRMS over a duration of nine years [20]. The EDSS score showed a decrease from a median of six to a median of 4.5.

Combined MS Stages

The observational study with the largest sample size (n=507) was conducted by Burt et al. in 2022 [21]. 81.7% of the sample were RRMS, and the remaining 18.3% were newly diagnosed with SPMS. The patients with RRMS reported final EDSS scores of 2.5 at year two and 2.33 at year four, whereas those with SPMS had EDSS scores at two and four years of 4.88 and 4.72, respectively. According to the authors, the data suggests that HSCT is effective for RRMS of an inflammatory nature, but to a lesser degree for SPMS, which is of a neurodegenerative nature.

Similar results were yielded in another study by Burt et al. in 2015 [22]. The four-year PFS and RFS were 87% and 80% for the entire population. At five years of follow-up, the median EDSS score changed from 4 to 2.5, and there was a significant reduction in the MRI disease activity. Additionally, treatment with HSCT was able to demonstrate a notable improvement in the quality of life, and cognitive, behavioral, and motor function.

Nicholas et al. demonstrated a decrease in the ARR in the overall population, with all the relapses occurring in the RRMS subgroup [23]. The EDSS score improvement was more notable in RRMS patients than those in the progressive group. After four years, 65% of the total population was free from disability progression (PFS), and NEDA-3 was 53%.

In the article by Muraro et al. a PFS of 46% at 5 years follow-up was reported [24]. The study showed that younger age, relapsing course of disease, and prior treatment with two or fewer immunosuppressive/modulatory treatments were associated with better progression-free survival.

Fassas et al. included patients most of whom were in the progressive phase with a high initial median EDSS score [25]. The overall PFS at the three-year follow-up was 74%. That of the subgroup PPMS was 66%, whereas the PFS of patients with SPMS or RRMS was 78%. Age less than 40 and stage not primary progressive were associated with higher PFS.

Mancardi et al. evaluated the long-term efficacy of HSCT [26]. After more than seven years of follow-up, EDSS score improvement was seen in 27% of patients, stabilization in 17% of patients, and progression in 56% of patients. Similarly, the PFS in this study was higher in individuals younger than 40 years of age, relapsing-remitting course, and those with gadolinium-enhancing lesions on baseline MRI.

A favorable PFS at five years’ follow-up was reported by Burman et al. [27]. At the final follow-up, PFS was 77%, RFS was 87%, and MRI-free survival was 85%. The calculated NEDA-3 was 68% at five years’ follow-up. Chen et al., despite the higher baseline EDSS score of included patients, has reported a 40% overall EDSS improvement 28% stabilization, and 32% worsening [28]. The study by Casanova et al. reported a 92% reduction in the ARR two years after HSCT. The evaluable NEDA-2 was 54.8%. After seven years of follow-up, the disability was improved in 60% of subjects and stabilized in 40% [29]. In the study by Krasulova et al. the overall PFS at three and six years was respectively 70.8% and 29.2% [30]. Better outcomes were observed in patients with a disease duration of less than five years and those younger than 35.

Häußler et al. compared the efficacy of HSCT with that of Alemtuzumab [31]. After one year, 35.7% of those who had received the transplant showed improvement in the EDSS score. The NEDA-3 was 60% for the HSCT group vs. 40% for the alemtuzumab group. In addition, this study has demonstrated significant cognitive improvement in the transplanted group. Das et al. demonstrated a NEDA-3 at 2.5 years of follow-up of 85%, with a significant reduction in the final median EDSS score and MRI activity [32]. The calculated PFS one year after HSCT was 100% in the small study by Dayama et al. [33]. Frau et al. followed nine patients for a median of 11 years, and reported a notable reduction in the number of relapses two years after transplant [34]. The final EDSS improved in 22.22%, stabilized in 22.22%, and worsened in 55.6%.

Safety and Feasibility of SCT in MS

A prospective phase 1 clinical trial (STEMS) aimed to assess the safety, feasibility, and tolerability of intrathecal administration of human fetal neural progenitor cells (hfNPCs) in patients with progressive multiple sclerosis (PMS) [35]. It provided valued information on the safety profile of hfNPC transplantation, and it demonstrated no significant improvements in neurologic and neurophysiological outcomes. However, it showed some interesting findings regarding brain volume changes and gray matter atrophy rates, which may warrant further investigation in larger cohorts.

An open-label prospective phase I/IIa clinical trial aimed to evaluate the safety, feasibility, and efficacy of using autologous bone marrow-derived mesenchymal stromal cells (BM-MSCs) followed by mesenchymal stromal cell-conditioned media (MSC-CM) for the treatment of multiple sclerosis (MS) patients [36]. The study also investigated the correlation between the content of MSC-CM and treatment outcomes. The treatment protocol was well tolerated by the patients, with no life-threatening adverse events reported. There was an overall trend of improvement in most of the tests, except for the lesion volume on MRI, which increased significantly. The treatment protocol was safe and feasible, with potential efficacy.

A retrospective single-center observational study of all MS patients (n=30) who underwent HSCT in Norway from January 2015 to January 2018 showed that 83% of patients achieved NEDA-3 status and none required any disease-modifying treatment after HSCT. However, long-term adverse events with amenorrhea prevailed in 43% of female patients [19].

A study published in October 2014 by Burman et al. illustrating the Swedish experience with autologous hematopoietic stem cell transplantation for aggressive multiple sclerosis revealed a five-year relapse-free survival in 87% of the patients [27]. The most common long-term side effects were herpes zoster reactivation (15%) and thyroid disease (8.4%).

A two-year follow-up of a phase I study to determine the long-term safety and efficacy of repeated intrathecal (IT) administration of autologous mesenchymal stem cell-derived neural progenitors (MSC-NPs) in patients with progressive MS showed no long-term adverse events associated with repeated IT-MSC-NP treatment [37]. However, the degree of disability reversal was not sustained in a subset of patients.

Burt et al. published in 2019 aiming to compare the effect of non-myeloablative HSCT versus disease-modifying therapy (DMT) on disease progression. This RCT revealed a prolonged time to disease progression in HSCT versus DMT [7].

An RCT published in 2023 by Petrou et al. provided clear signals of short-term clinical efficacy and possible indications of neuroprotection, induced by the administration of autologous MSCs in patients with progressive multiple sclerosis [38]. The findings suggest a superiority of intrathecal over intravenous administration and indicate a possible boost of the beneficial effects by repeated injection of the cells.

In 2021, the analysis of long-term outcomes after aHSCT in 210 patients of whom 58% relapsing-remitting multiple sclerosis (RRMS) showed that aHSCT prevents disability worsening in the majority of patients and induces durable improvement in disability in patients with RRMS [39].

To evaluate the long-term outcomes in patients who underwent aHSCT for the treatment of MS in a large multicenter cohort, Muraro et al. obtained data from 25 centers in 13 countries. In this observational study of patients with MS treated with aHSCT, almost half of them remained free from neurological progression for five years after transplant. Younger age, relapsing form of MS, fewer prior immunotherapies, and lower baseline EDSS score were factors associated with better outcomes. The results support the rationale for further randomized clinical trials of aHSCT for the treatment of MS.

Discussion

The study's literature search process and selection criteria led to the inclusion of 37 relevant articles, including RCTs, and observational, retrospective, and comparative studies. The studies evaluated various aspects of SCT in different stages of MS, predominantly RRMS and SPMS. The results highlight the potential benefits and limitations of SCT as a therapeutic approach for MS.

Patients treated with HSCT present lower progression and relapse rates, suppression of inflammatory activity, and a greater reduction in T2 lesions on MRI than those treated with DMTs. Non-myeloablative chemotherapy was shown to be more efficient than myeloablative aHSCT due to a higher disease progression rate in the latter. This finding was also confirmed by an observational, retrospective study on RRMS patients that also showed better survival in patients receiving non-myeloablative therapy.

HSCT showed better outcomes than fingolimod and alemtuzumab but not natalizumab. Furthermore, patients treated with HSCT had an earlier and faster recovery from disabilities. HSCT showed a reduction in ARR with no MRI activity in RRMS. HSCT was able to demonstrate a notable improvement in the quality of life, and cognitive, behavioral, and motor function. HSCT was also proven to be effective for patients with aggressive MS early in their disease course by improving EDSS score and removing MRI activity in the majority of these patients.

A more important improvement of EDSS was noted in RRMS than in progressive MS and shows that HSCT is more efficient in treating the inflammatory nature of the disease than the neurodegenerative one. Additionally, EDSS has a higher probability of worsening in progressive MS. EDSS keeps improving even four years after the treatment but improvement becomes slower.

Factors that enhance HSCT/ aHSCT efficiency and increase PFS include: younger age (<35-40), relapsing rather than progressive -especially primary progressive- course of the disease, the presence, at baseline, of gadolinium-enhancing lesions on MRI and fewer prior immunosuppressive treatments (two or less), lower baseline EDSS score

Compared to DMT, treatment with HSCT shows a prolonged time for disease progression to reappear. Some HSCT long-term adverse events were identified such as amenorrhea (43%), Herpes zoster reactivation (45%), and thyroid disease (8%). However, bigger studies are needed to confirm the prevalence of these adverse events and investigate additional possible side effects.

Intrathecal administration of autologous MSCs presents better outcomes than an intravenous administration. Moreover, repeated Intrathecal injection was confirmed to be safe and has a potential boosting effect that’s worth being further investigated.

Effectiveness of SCT in MS

The findings indicate that SCT has demonstrated efficacy in reducing disease activity and progression in MS patients. The evidence is primarily derived from RCTs, observational, retrospective, and comparative studies. Two randomized controlled trials (RCTs) are discussed, namely the ASTIMS trial and the MIST trial. Despite methodological limitations, both trials suggest that SCT can significantly reduce the appearance of new T2 lesions on MRI and suppress inflammatory activity. However, the improvement in disability scores varied between studies.

Observational and Retrospective Studies

A substantial portion of the findings comes from observational and retrospective studies. These studies report outcomes across various MS subtypes, such as RRMS and SPMS. They provide evidence of SCT's potential to halt disease progression, reduce relapse rates, and improve disability scores. The studies show favorable results, including the reduction of annualized relapse rates (ARR), improved Expanded Disability Status Scale (EDSS) scores, and high proportions of patients achieving no evidence of disease activity (NEDA).

Safety and Feasibility

The discussion also addresses the safety and feasibility of SCT in MS treatment. Several studies evaluated the safety profile of different SCT approaches, including intrathecal administration of neural progenitor cells and autologous bone marrow-derived mesenchymal stromal cells (BM-MSCs). While some studies reported positive safety outcomes, others highlighted potential adverse events, such as herpes zoster reactivation and thyroid disease. Long-term effects, especially in female patients, were also mentioned.

Considerations and limitations

The findings underscore the potential of SCT as a treatment option for MS, particularly in reducing disease activity and progression. However, there are several limitations to consider. Many of the studies have small sample sizes, potentially affecting the generalizability of the results. Variability in study designs, SCT protocols, and patient populations further complicates direct comparisons. Additionally, the discussion points out that certain SCT trials excluded specific disease-modifying therapies (DMTs) due to FDA approvals and safety concerns at the time of enrollment.

Clinical implications and future directions

Overall, the findings suggest that SCT may hold promise for specific subsets of MS patients, particularly those with RRMS, but its effectiveness for progressive forms of the disease is less clear. The discussion highlights the need for further research, including larger and well-controlled trials, to better understand the long-term effects, potential benefits, and risks associated with SCT in MS treatment. It emphasizes the importance of considering patient characteristics, disease stage, and individual response when evaluating the suitability of SCT as a therapeutic option.

More RCTs are needed with a bigger sample size tackling more precisely each phenotype of the disease and including more FDA-approved DMTs. Studies aiming to clearly determine HSCT’s adverse effects in order to be compared with DMTs’ adverse events so as to have a clearer picture, be able to more accurately determine the best treatment course, and try to improve treatment so as to minimize unwanted side effects.

## Conclusions

This review highlights the potential of SCT as a therapeutic approach for multiple sclerosis (MS). The evidence is drawn from a range of studies, including RCTs, observational, retrospective, and comparative studies. SCT has shown promise in reducing disease activity and progression, especially in RRMS. While RCTs suggest SCT can reduce new lesions and inflammation, the impact on disability scores varies.

Observational and retrospective studies support SCT's potential, showing reduced relapses, improved disability scores, and a significant number of patients achieving NEDA. However, the discussion on safety and feasibility is nuanced. Studies on alternative SCT approaches, like intrathecal administration of neural progenitor cells and autologous BM-MSCs, offer insights into safety but also note potential adverse events.

The review calls for more comprehensive, large-scale studies to provide clearer insights into SCT's potential across different MS forms. It emphasizes that SCT's application should consider individual patient characteristics, disease stages, and responses. While SCT holds promise for MS treatment, further research is needed to understand its benefits, risks, and long-term effects. These findings serve as a foundation for refining MS treatment strategies and shaping the future of MS management.

## References

[1] Tafti D, Ehsan M, Xixis KL (2023). Multiple sclerosis. StatPearls [Internet].

[2] Coyle PK (2021). What can we learn from sex differences in MS?. J Pers Med.

[3] Walton C, King R, Rechtman L (2020). Rising prevalence of multiple sclerosis worldwide: insights from the atlas of MS, third edition. Mult Scler.

[4] Hrishi AP, Sethuraman M (2019). Cerebrospinal fluid (CSF) analysis and interpretation in neurocritical care for acute neurological conditions. Indian J Crit Care Med.

[5] Khaddour K, Hana CK, Mewawalla P (2023). Hematopoietic stem cell transplantation. StatPearls [Internet].

[6] Mancardi GL, Sormani MP, Gualandi F (2015). Autologous hematopoietic stem cell transplantation in multiple sclerosis: a phase II trial. Neurology.

[7] Burt RK, Balabanov R, Burman J (2019). Effect of nonmyeloablative hematopoietic stem cell transplantation vs continued disease-modifying therapy on disease progression in patients with relapsing-remitting multiple sclerosis: a randomized clinical trial. JAMA.

[8] Boffa G, Signori A, Massacesi L (2023). Hematopoietic stem cell transplantation in people with active secondary progressive multiple sclerosis. Neurology.

[9] Mariottini A, Bulgarini G, Forci B (2022). Autologous haematopoietic stem cell transplantation versus low-dose immunosuppression in secondary-progressive multiple sclerosis. Eur J Neurol.

[10] Mariottini A, Filippini S, Innocenti C (2021). Impact of autologous haematopoietic stem cell transplantation on disability and brain atrophy in secondary progressive multiple sclerosis. Mult Scler.

[11] Jespersen F, Petersen SL, Andersen P, Sellebjerg F, Magyari M, Sørensen PS, Blinkenberg M (2023). Autologous hematopoietic stem cell transplantation of patients with aggressive relapsing-remitting multiple sclerosis: Danish nation-wide experience. Mult Scler Relat Disord.

[12] Kalincik T, Sharmin S, Roos I (2023). Comparative effectiveness of autologous hematopoietic stem cell transplant vs fingolimod, natalizumab, and ocrelizumab in highly active relapsing-remitting multiple sclerosis. JAMA Neurol.

[13] Zhukovsky C, Sandgren S, Silfverberg T (2021). Autologous haematopoietic stem cell transplantation compared with alemtuzumab for relapsing-remitting multiple sclerosis: an observational study. J Neurol Neurosurg Psychiatry.

[14] Boffa G, Lapucci C, Sbragia E (2020). Aggressive multiple sclerosis: a single-centre, real-world treatment experience with autologous haematopoietic stem cell transplantation and alemtuzumab. Eur J Neurol.

[15] Kvistad SA, Lehmann AK, Trovik LH, Kristoffersen EK, Bø L, Myhr KM, Torkildsen Ø (2020). Safety and efficacy of autologous hematopoietic stem cell transplantation for multiple sclerosis in Norway. Mult Scler.

[16] Giedraitiene N, Kizlaitiene R, Peceliunas V, Griskevicius L, Kaubrys G (2020). Selective cognitive dysfunction and physical disability improvement after autologous hematopoietic stem cell transplantation in highly active multiple sclerosis. Sci Rep.

[17] Mariottini A, Innocenti C, Forci B (2019). Safety and efficacy of autologous hematopoietic stem-cell transplantation following natalizumab discontinuation in aggressive multiple sclerosis. Eur J Neurol.

[18] Tolf A, Fagius J, Carlson K, Åkerfeldt T, Granberg T, Larsson EM, Burman J (2019). Sustained remission in multiple sclerosis after hematopoietic stem cell transplantation. Acta Neurol Scand.

[19] Kvistad SA, Burman J, Lehmann AK (2022). Impact of previous disease-modifying treatment on safety and efficacy in patients with MS treated with AHSCT. J Neurol Neurosurg Psychiatry.

[20] Comini-Frota ER, Marques BC, Torres C, Cohen KM, Miranda EC (2019). Nine-year follow up after hematopoietic stem cell transplantation in five multiple sclerosis patients. Arq Neuropsiquiatr.

[21] Burt RK, Han X, Quigley K, Helenowski IB, Balabanov R (2022). Real-world application of autologous hematopoietic stem cell transplantation in 507 patients with multiple sclerosis. J Neurol.

[22] Burt RK, Balabanov R, Han X (2015). Association of nonmyeloablative hematopoietic stem cell transplantation with neurological disability in patients with relapsing-remitting multiple sclerosis. JAMA.

[23] Nicholas RS, Rhone EE, Mariottini A (2021). Autologous hematopoietic stem cell transplantation in active multiple sclerosis: a real-world case series. Neurology.

[24] Muraro PA, Pasquini M, Atkins HL (2017). Long-term outcomes after autologous hematopoietic stem cell transplantation for multiple sclerosis. JAMA Neurol.

[25] Fassas A, Passweg JR, Anagnostopoulos A (2002). Hematopoietic stem cell transplantation for multiple sclerosis. A retrospective multicenter study. J Neurol.

[26] Mancardi GL, Sormani MP, Di Gioia M (2012). Autologous haematopoietic stem cell transplantation with an intermediate intensity conditioning regimen in multiple sclerosis: the Italian multi-centre experience. Mult Scler.

[27] Burman J, Iacobaeus E, Svenningsson A (2014). Autologous haematopoietic stem cell transplantation for aggressive multiple sclerosis: the Swedish experience. J Neurol Neurosurg Psychiatry.

[28] Chen B, Zhou M, Ouyang J (2012). Long-term efficacy of autologous haematopoietic stem cell transplantation in multiple sclerosis at a single institution in China. Neurol Sci.

[29] Casanova B, Jarque I, Gascón F (2017). Autologous hematopoietic stem cell transplantation in relapsing-remitting multiple sclerosis: comparison with secondary progressive multiple sclerosis. Neurol Sci.

[30] Krasulová E, Trneny M, Kozák T (2010). High-dose immunoablation with autologous haematopoietic stem cell transplantation in aggressive multiple sclerosis: a single centre 10-year experience. Mult Scler.

[31] Häußler V, Ufer F, Pöttgen J (2021). aHSCT is superior to alemtuzumab in maintaining NEDA and improving cognition in multiple sclerosis. Ann Clin Transl Neurol.

[32] Das J, Snowden JA, Burman J (2021). Autologous haematopoietic stem cell transplantation as a first-line disease-modifying therapy in patients with 'aggressive' multiple sclerosis. Mult Scler.

[33] Dayama A, Bhargava R, Kurmi SR, Jain S, Dua V (2020). Autologous stem cell transplant in adult multiple sclerosis patients: a study from North India. Neurol India.

[34] Frau J, Carai M, Coghe G (2018). Long-term follow-up more than 10 years after HSCT: a monocentric experience. J Neurol.

[35] Genchi A, Brambilla E, Sangalli F (2023). Neural stem cell transplantation in patients with progressive multiple sclerosis: an open-label, phase 1 study. Nat Med.

[36] Dahbour S, Jamali F, Alhattab D (2017). Mesenchymal stem cells and conditioned media in the treatment of multiple sclerosis patients: clinical, ophthalmological and radiological assessments of safety and efficacy. CNS Neurosci Ther.

[37] Harris VK, Stark JW, Yang S, Zanker S, Tuddenham J, Sadiq SA (2021). Mesenchymal stem cell-derived neural progenitors in progressive MS: two-year follow-up of a phase I study. Neurol Neuroimmunol Neuroinflamm.

[38] Petrou P, Kassis I, Levin N (2020). Beneficial effects of autologous mesenchymal stem cell transplantation in active progressive multiple sclerosis. Brain.

[39] Boffa G, Massacesi L, Inglese M (2021). Long-term clinical outcomes of hematopoietic stem cell transplantation in multiple sclerosis. Neurology.

